# Comparison of Clinical Outcomes After Cataract Surgery with Implantation of Either a Partial-Depth of Field Extended or Monofocal Intraocular Lens

**DOI:** 10.3390/jcm15020830

**Published:** 2026-01-20

**Authors:** Helena Noguera, Ignacio Gutiérrez Santamaría, Iñaki Basterra, Sergio Díaz Gómez, Angelica Pérez, Gorka Lauzirika, David P. Piñero

**Affiliations:** 1 Centro Oftalmológico Integral Bilbao Berri SL (Miranza COI Bilbao), 48008 Bilbao, Spain; helena.noguera@miranza.es (H.N.); ignacio.gutierrez@miranza.es (I.G.S.);; 2 R&D Department of Miranza Group, 08035 Barcelona, Spain; 3 Miranza Begitek, 20012 Donostia-San Sebastián, Spain; 4 Department of Optics, Pharmacology and Anatomy, University of Alicante, 03690 Alicante, Spain

**Keywords:** partial depth of field IOL, extended depth of focus, spectacle independence, defocus curve, intermediate visual acuity, functional vision

## Abstract

**Background/Objectives**: To compare the clinical outcomes following cataract surgery with implantation of a new partial depth of field (DOFi) intraocular lens (IOL) versus a monofocal IOL of identical material, platform, and haptic design. **Methods**: Single-center, non-randomized trial including 55 patients randomly assigned to be implanted either with the partial-DOFi IOL Tecnis PureSee (partial-DOFi group, 29 patients) or the Tecnis monofocal IOL DCB00/ZCU (both Johnson & Johnson Surgical Vision) (monofocal group, 26 patients). Monocular visual acuity (VA), refractive, binocular defocus curve, and patient-reported outcomes (QoV and Catquest 9SF questionnaires) were evaluated during a 3-month follow-up. **Results**: No significant differences between monofocal and partial-DOFi groups were found in monocular postoperative uncorrected- (0.03 ± 0.08 vs. 0.05 ± 0.10, *p* = 0.419) and corrected-distance VA (−0.03 ± 0.04 vs. −0.03 ± 0.05, *p* = 0.642). Significantly better distance-corrected intermediate VA was found in the partial-DOFi group (0.29 ± 0.08 vs. 0.10 ± 0.06, *p* < 0.001). Similarly, postoperative monocular distance-corrected near VA was better in the partial-DOFi group (0.51 ± 0.10 vs. 0.31 ± 0.09, *p* < 0.001). In the defocus curve, significantly better distance-corrected VAs compared to monofocal were found for all defocus levels from −1.50 to −4.00 D. Minor reports of starbursts were found in both IOL groups. With the Catquest questionnaire, some significant differences were found between groups including reduced difficulty reading newspapers (*p* < 0.001), improved visibility of prices while shopping (*p* < 0.001) and enhanced performance of hobbies (*p* = 0.030) and needlework (*p* < 0.001). **Conclusions**: The partial-DOFi IOL evaluated demonstrates superior intermediate and near visual performance compared to a monofocal IOL, while maintaining equivalent distance vision and visual quality.

## 1. Introduction

A wide range of intraocular lens (IOL) technologies has been developed in recent years, each offering distinct levels of visual performance through different optical principles [1]. Indeed, some lenses marketed as extended depth-of-focus IOLs may show bifocal or low-add functional behavior on defocus curves which would strengthen the conceptual framing [1]. In response to this diversity, the European Society of Cataract and Refractive Surgeons (ESCRS) established a new functional classification system through expert consensus, aiming to help clinicians understand the expected visual rehabilitation outcomes for each lens type [2,3]. This classification system distinguishes between full- and partial- depth of field (DOFi) IOLs based on the functional visual field demonstrated in monocular defocus curves with best distance correction at 0.2 logMAR. Partial-DOFi IOLs are defined as those providing less than 2.3 diopters (D) of depth of field and less than 2.75 D at the 0.3 LogMAR [2,3]. Furthermore, partial-DOFi IOLs are subcategorized according to the following: narrow when the depth of field (DoFi) in 0.2 logMAR is below 1.2 D and DoF in 0.3 logMAR < 1.61 D, enhance when DoFi in 0.2 logMAR (D) ≥ 1.2 and <1.58 and DoFi in 0.3 logMAR (D) ≥ 1.61 and <1.98, and extend when DoFi in 0.2 logMAR (D) ≥ 1.58 and <2.3 and DoFi in 0.3 logMAR (D) ≥ 1.98 and <2.75 [2,3].

Numerous partial-DOFi IOL designs have been developed with the common goal of extending the eye’s depth of field while minimizing adverse visual effects such as photic phenomena or reduced contrast sensitivity [2,3,4,5]. These lenses employ various optical approaches, either individually or in combination, including manipulation of spherical aberration (positive or negative), pinhole effect utilization, low-add multifocal designs (refractive or diffractive), central zone geometry modifications creating differential refractive power distribution, and wavefront modulation [1]. Recently, the TECNIS PureSee IOL (Johnson & Johnson Surgical Vision, Irvine, CA, USA) has been introduced as a novel partial-DOFi design. This lens features a continuous refractive power transition achieved through a modified posterior surface curvature that progressively distributes light from far through intermediate-to-near vision while maintaining the same anterior surface aspherical design like all the other TECNIS IOLs [6]. Optical bench studies have characterized this IOL’s performance [7,8,9], while clinical investigations have demonstrated its extended range of vision [8] and tolerance to residual refractive errors [10].

The present study aims to compare visual, refractive, photic effects, and defocus curve outcomes following cataract surgery with implantation of this new partial-DOFi IOL versus a monofocal IOL of identical material, platform, and haptic design and to evaluate if this IOL meets the American National Standard Z80.35–2018 (ANSI) of extended depth-of-focus (EDOF) IOLs [11] as well as the criteria for being classified as extended partial-DOFi according to the new classification of the ESCRS [2,3].

## 2. Materials and Methods

This single-center, non-randomized clinical trial was conducted at Miranza Bilbao (Spain). Patients scheduled for routine bilateral cataract surgery were enrolled, including one group receiving the partial-DOFi IOL Tecnis PureSee model DEENV00/DET, and another one the monofocal IOL Tecnis model DCB00/ZCU (both from Johnson & Johnson Surgical Vision, Irvine, CA, USA). Inclusion criteria required patients to have cataractous eyes without significant ocular comorbidity, be aged ≥50 years, and provide written informed consent and demonstrate willingness to complete all study procedures. Exclusion criteria included degenerative visual disorders (e.g., macular degeneration, retinal/optic nerve pathology), prior intraocular/corneal surgery or intravitreal injections, history or presence of macular edema, glaucoma, amblyopia, corneal disorders such as keratoconus, chronic uveitis, and zonular instability/defect.

All patients provided written informed consent before enrolment. The study was approved by the ethics committee of Instituto de Microcirugía Ocular (IMO) (approval code: IMO 240520-261; approval date: 25 June 2024).

### 2.1. Clinical Protocol

A complete preoperative assessment was performed for all participants, including monocular uncorrected- (UDVA) and corrected-distance visual acuity (CDVA) measurements using ETDRS charts, manifest refraction, corneal topography, optical biometry (IOLMaster 700, Carl Zeiss Meditec, Jena, Germany), slit-lamp examination, pupillometry, Goldmann applanation tonometry, and fundus examination. Postoperatively, patients were evaluated on day 1, as well as at 1 and 3 months after surgery. On postoperative day 1, assessments included monocular UDVA, Goldmann applanation tonometry, and slit-lamp examination. At the 1-month follow-up, patients underwent manifest refraction, monocular UDVA and CDVA measurements, and monocular distance-corrected intermediate (DCIVA) and near visual acuity (DCNVA) assessments at 66 cm and 40 cm, respectively, along with slit-lamp anterior segment examination.

At the 3-month postoperative visit, in addition to the standard 1-month examination protocol, patients underwent monocular defocus curve testing with best distance-corrected refraction and spherical additions ranging from −4.0 D to +1.0 D in 0.5-D steps using the defocus curve app Multifocal Lens Analyzer (version 3.0) (Qvision Academy, Almería, Spain) under photopic conditions (85 cd/m^2^) [12], an assessment of photic phenomena perception using the validated Quality of Vision (QoV) questionnaire [13] and an evaluation of daily-life activity difficulties and overall satisfaction with visual outcomes using the Catquest-9SF questionnaire [14].

### 2.2. Intraocular Lenses

The Tecnis PureSee (DEN00V/DET) IOL is a purely refractive, single-piece, foldable presbyopia-correcting IOL made of soft hydrophobic acrylic (Sensar UV, OptiBlue, Johnson & Johnson Surgical Vision, Irvine, CA, USA) with an Abbe number of 55 and a refractive index of 1.47 (35 °C) [6,7]. It features continuous-power technology, combining an anterior aspheric surface (correcting average corneal spherical aberration) and a posterior refractive surface (extending depth of focus via continuous power change) [6,7]. It has an overall diameter of 13 mm and an optic zone of 6 mm. The IOL is available in spherical powers from +5.0 to +34.0 D (0.5 D increments) and in cylinder powers of 1.00, 1.50, 2.25, 3.00, 3.75, 4.50, 5.25, and 6.00 D (Tecnis PureSee Toric II DET). The lens comes preloaded in the TECNIS Simplicity™ Delivery System injector.

The Tecnis DCB00 is a single-piece preloaded monofocal IOL sharing the same hydrophobic acrylic material (Sensar UV) and platform as the PureSee lens. This biconvex lens features an anterior aspheric surface designed to compensate for average corneal spherical aberration, with an overall diameter of 13 mm and a 6.0 mm optic. A toric version (Tecnis Toric II ZCU) is available, incorporating posterior surface toricity for astigmatism correction.

### 2.3. Surgery

Two experienced surgeons (HN and IGS) performed all procedures using standardized sutureless microincision phacoemulsification. Each surgery began with the instillation of topical anesthetic and mydriatic drops to prepare the eye. After this preparation, the surgeons first created a 2.2 mm temporal clear corneal incision. Under the operating microscope, they then carefully fashioned a manual continuous curvilinear capsulorhexis with an intended diameter of 5.0 mm. The phacoemulsification procedure commenced, with thorough removal of lens material from the capsular bag. With the crystalline lens removed, the surgeons implanted the IOL into the capsular bag using the preloaded TECNIS Simplicity™ Delivery System injector (Johnson & Johnson Surgical Vision, Irvine, CA, USA). Before concluding, they verified proper IOL positioning and axis alignment using the Callisto eye-tracking system (Carl Zeiss Meditec. Jena, Germany) for optimal refractive outcomes. Postoperatively, patients were prescribed a combined antibiotic–steroid eye drop regimen to be administered four times daily for one week to support healing and prevent infection.

For all cases, IOL power calculations were performed using the Barrett formula, with an A-constant of 119.30 and the first myopic option considered as the refractive target.

### 2.4. Statistical Analysis

The required sample size was determined based on monocular DCIVA. Using data from Pedrotti et al. [15], which reported a postoperative DCIVA standard deviation of 0.2 logMAR, 30 subjects per group were found that would provide adequate power to detect inter-visit differences ≥ 0.10 logMAR between the 1-month and 3-month follow-ups. This calculation assumed a two-sided α = 0.05, β = 0.2 (80% power), and accounted for potential 10% attrition (anticipated in this prospective design).

All analyses were performed using SPSS v22.0 (IBM Corp., Armonk, NY, USA). The Kolmogorov–Smirnov test assessed data normality. Continuous variables are reported as mean ± standard deviation (range), while categorical variables are presented as frequencies (percentages). For intergroup comparisons, the unpaired Student *t* test was used for normally distributed data and the Mann–Whitney U test for non-normally distributed data. Chi-square test was used to assess the significance of differences in categorical variables. Statistical significance was set at *p* < 0.05.

Visual and refractive outcomes were analyzed and presented following the standardized cataract surgery reporting guidelines established by Reinstein et al. [16]. Although all surgeries were bilateral, only one eye for each patient was considered for the analysis to avoid the bias associated with the inclusion of data from both eyes. For the Catquest-9SF questionnaire, responses were converted to Rasch-calibrated scores using a 4-category Andrich rating scale, as per the original developers’ methodology [14]. Notably, higher scores on this scale indicate greater difficulty performing vision-related tasks and lower patient satisfaction with visual outcomes.

Finally, four effectiveness endpoints should be met in full according to the ANSI Standard Z80.35–2018 for classifying an IOL as EDOF and all of them were analyzed and confirmed if they were met [11]:

1. A statistical superiority over a control monofocal group on mean monocular DCIVA at 66 cm.
2. The monocular DoF for the EDOF-implanted eyes needs to be at least 0.5 D greater than the DoF for the monofocal IOL controls at logMAR 0.2.
3. The EDOF IOL needs to have at least 50% of eyes achieving monocular DCIVA of better than or equal to 0.2 logMAR at 66 cm.
4. Mean monocular CDVA for the EDOF IOL is not statistically inferior to the control using a non-inferiority margin of 0.1 logMAR.

Only one eye per patient was randomized for analysis, using a random number sequence, to avoid bias associated with the interocular correlation.

## 3. Results

Fifty-five patients were bilaterally implanted in the study. A total of 26 (47.3%) patients were implanted with the monofocal IOL and 29 (52.7%) with the partial-DOFi IOL. A total of 27 right eyes (49.1%) and 28 left eyes (50.9%) were evaluated. Preoperative baseline characteristics are summarized in Table 1. Statistically significant intergroup differences were only observed in age (*p* = 0.009). However, no significant differences were found in mean spherical (monofocal: 19.46 ± 4.07 D; partial-DOFi: 19.69 ± 5.94 D; *p* = 0.179) and cylinder IOL power (monofocal: 0.43 ± 0.96 D; partial-DOFi: 0.58 ± 0.94 D; *p* = 0.408). Likewise, no significant differences between groups were present in predicted postoperative spherical equivalent (SE) (monofocal: −0.27 ± 0.17 D; partial-DOFi: −0.29 ± 0.23 D; *p* = 0.393). Toric IOLs were implanted in five eyes (19.2%) in the monofocal group and eight eyes (27.6%) in the partial-DOFi group.

### 3.1. Visual and Refractive Outcomes

**Figure 1:**
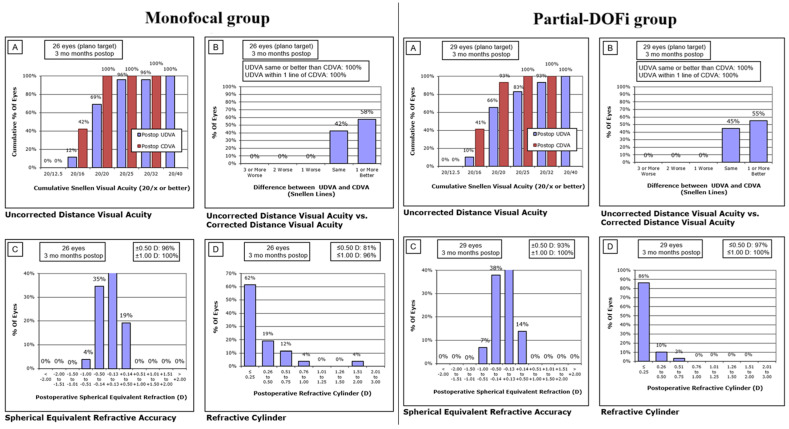
Graphic reporting of outcomes of cataract surgery in the two groups evaluated according to the guidelines defined by Reinstein et al. [16]. (**A**) Cumulative uncorrected distance visual acuity (UDVA) at different Snellen lines (e.g., 20/20, 20/32, 20/40). (**B**) Distribution of differences in UDVA and Corrected Distance Visual Acuity (CDVA) in Snellen lines. (**C**) Distribution of postoperative spherical equivalent (SE) refraction. (**D**) Distribution of postoperative refractive cylinder.

Postoperative monocular distance visual and refractive outcomes are presented in Figure 1. At the 3-month follow-up, the spherical equivalent (SE) was within ±1.00 D in all eyes from the monofocal and partial-DOFi groups, respectively (Figure 1). Similarly, the SE fell within ±0.50 D in 96% (monofocal) and 97% (partial-DOFi) of cases. Residual cylinder ≤ 1.00 D was observed in 96% (monofocal) and 100% (partial-DOFi) of eyes at 3 months postoperatively.

Table 2 summarizes postoperative visual and refractive outcomes in the two IOL groups evaluated. Significant intergroup differences were observed in DCIVA and DCNVA at 1 and 3 months after surgery, with better values in the partial-DOFi group (*p* < 0.001). Likewise, 3-month postoperative cylinder was significantly lower in the partial-DOFi group compared to monofocal (*p* = 0.035). In the initial postoperative period (1 week after surgery), significant differences between groups were found in sphere (*p* = 0.014) and SE (*p* = 0.020).

**Figure 2:**
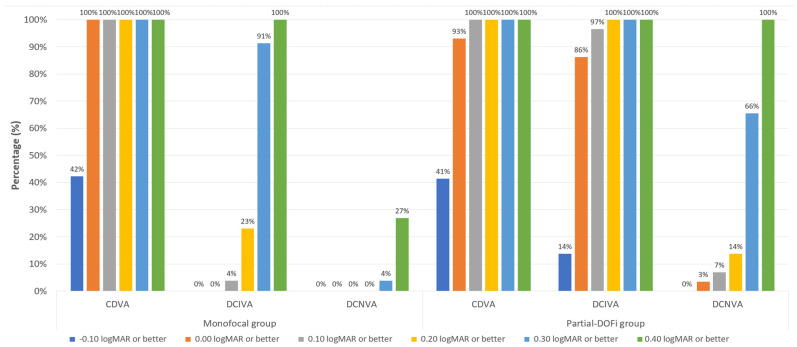
Distribution of corrected binocular 3-month postoperative visual acuity data in the two groups of the sample evaluated. Abbreviations: CDVA, corrected-distance visual acuity; DCIVA, distance-corrected intermediate visual acuity; DCNVA, distance-corrected near visual acuity.

For 3-month postoperative monocular CDVA, a total of 100% (monofocal) and 93% (partial-DOFi) achieved a monocular CDVA of 0.00 logMAR or better (Figure 2). Postoperative DCIVA at 3 months was ≤0.20 logMAR in 23% (monofocal) and 100% (partial-DOFi) of patients (Figure 2). Finally, concerning 3-month postoperative DCNVA, 4% (monofocal) and 66% (partial-DOFi) of eyes achieved a value of 0.30 logMAR or better (Figure 2).

### 3.2. Defocus Curve Outcomes

**Figure 3:**
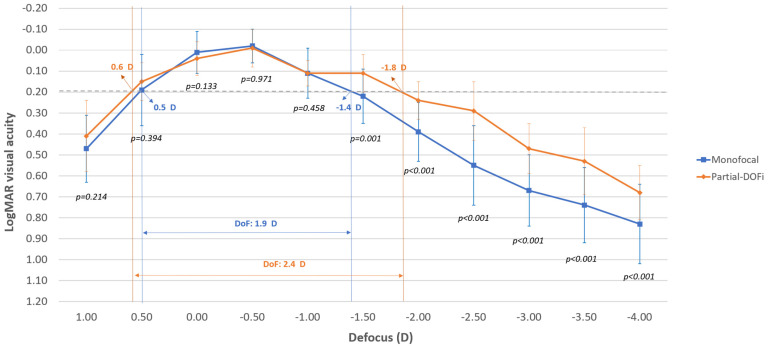
Mean postoperative monocular defocus curve in the two groups of the sample evaluated. The dotted line represents the visual acuity value 0.20 logMAR.

Figure 3 displays the mean postoperative monocular defocus curves for the two IOL groups. Significantly better distance-corrected visual acuities were found in the partial-DOFi group compared to monofocal for all defocus levels from −1.50 to −4.00 D (*p* ≤ 0.001). The monofocal IOL maintained mean distance-corrected visual acuity ≤ 0.30 logMAR across defocus levels from +0.50 D to −1.50 D (Figure 3). However, the visual acuity was maintained within this range with the partial-DOFi IOL for defocus levels between +0.50 D and −2.50 D (Figure 3).

### 3.3. Patient-Reported Outcomes

Concerning the QoV questionnaire, only starbursts were reported by patients, with two subjects (7.7%) perceiving them in the monofocal group and three (10.3%) in the partial-DOFi group though they were not bothersome.

Table 3 compares the 3-month postoperative Rasch-calibrated scoring obtained with the Catquest-9SF questionnaire in the two groups of the sample evaluated. As shown, statistically significant differences between IOL groups in the postoperative Rasch-calibrated scores were found in item A (*p* = 0.002), item C1 (*p* < 0.001), item C3 (*p* < 0.001), item C5 (*p* < 0.001), and item C7 (*p* = 0.030). According to this, less difficulties were found in the partial-DOFi group for performing activities such as reading text in the newspaper, seeing prices of goods when shopping, seeing to do needlework and handicraft and seeing to carry out a preferred hobby.

### 3.4. Analysis of EDOF and Extend Partial-DOFi Criteria

Concerning the ANSI Standard Z80.35–2018 for classifying an IOL as EDOF, the four critical effectiveness endpoints were reached:

1. Significantly better DCIVA (66 cm) at 1 and 3 months after surgery with the partial-DOFi IOL evaluated compared to a monofocal IOL (*p* < 0.001).
2. According to the monocular defocus curve, the DoF at 0.2 logMAR for eyes implanted with the partial-DOFi IOL evaluated was 0.5 D greater than the DoF measured in eyes with the monofocal IOL (Figure 3).
3. All eyes implanted with the partial-DOFi IOL achieved a 3-month postoperative monocular DCIVA of 0.2 logMAR or better.
4. No statistically significant differences were found between the partial-DOFi and monofocal IOLs evaluated in 3-month postoperative CDVA (*p* = 0.642).

Concerning the ESCRS functional classification system, the TECNIS PureSee IOL can be considered as a partial-DOFi lens as it provides a DoF at 0.2 logMAR of 2.4 D, close to the upper limit for DoF corresponding to partial-DOFi IOLs (DoF < 2.3 D). Furthermore, DoF at 0.3 logMAR is around 3.00 D, close to the maximum limit of 2.75 D for being considered a partial-DOFi IOL. Likewise, the partial-DOFi IOL can subcategorized as extend because the DoF in 0.2 and 0.3 logMAR was within the range defined for this subcategory (Figure 3).

## 4. Discussion

This prospective comparative study evaluated the clinical outcomes of a new partial-DOFi IOL and compared them with those of a conventional monofocal IOL of the same material and platform. Both lenses demonstrated excellent distance visual outcomes, with no significant differences between the partial-DOFi and monofocal IOLs in terms of 3-month postoperative UDVA (0.03 ± 0.08 vs. 0.05 ± 0.10, *p* = 0.419) and CDVA (−0.03 ± 0.04 vs. −0.03 ± 0.05, *p* = 0.642). The performance of the evaluated partial-DOFi IOL was similar [17,18,19] or even better than that reported for other partial-DOFi IOL models [20,21,22]. This comparison with previous studies of other partial-DOF IOLs, which have different designs (refractive or diffractive) and materials, confirms that the evaluated partial-DOF IOL provides visual and refractive results comparable to, or better than, other commercially available models also categorized as partial-DOFi lenses. For example, Obergozo et al. [20] evaluated the clinical outcomes of a refractive partial-DOFi IOL (LuxSmart, Bausch & Lomb), reporting mean 3-month postoperative UDVA and CDVA values of 0.10 ± 0.11 and 0.03 ± 0.08 logMAR, respectively. Similarly, Rabinovich et al. [18] reported in another trial assessing a different partial-DOFi IOL (Lucidis IOL, Swiss Advanced Vision) a mean 3-month postoperative monocular UDVA and CDVA of 0.067 ± 0.08 and 0.028 ± 0.04 logMAR, respectively. Schojai et al. [22] found in another comparative study that a diffractive-based partial-DOFi IOL (Symfony, Johnson & Johnson Vision) provided a mean 3-month postoperative monocular UDVA of 0.11 ± 0.11 logMAR.

The excellent uncorrected UDVA outcomes observed with the evaluated partial-DOFi were consistent with its high refractive predictability. Specifically, 97% of eyes achieved a 3-month postoperative SE within ±0.50 D, and 100% had residual cylinder ≤ 1.00 D. These results were comparable to those of the monofocal IOL, with only a minor (mean difference of 0.21 D) but statistically significant difference in the refractive cylinder at 3 months postoperatively. Specifically, the partial-DOFi demonstrated a lower magnitude of residual cylinder, supporting its efficacy in astigmatism correction. However, it should be noted that more toric IOLs were implanted in the partial-DOFi group, which may have influenced the results. In addition, the mean difference fell within the accepted manufacturing tolerance range for toric IOL cylinder, suggesting that its clinical relevance is limited. Further studies using vector analysis are warranted to better assess the astigmatic correction efficacy of this IOL.

The partial-DOFi extend demonstrated significantly better DCIVA than the monofocal IOL. Specifically, the mean 3-month postoperative monocular DCIVA was 0.10 ± 0.06 logMAR with the partial-DOFi IOL versus 0.29 ± 0.08 logMAR with the monofocal IOL (*p* < 0.001), confirming the partial-DOFi IOL’s superior ability to restore intermediate vision. Likewise, this significant difference in DCIVA compared to the monofocal IOL and the achievement of DIVA of 0.20 logMAR in all cases confirms the behavior of the TECNIS PureSee IOL as an EDOF IOL. These results align with [17,18,20] and even exceed [19,21,23,24], i.e., those reported for other partial-DOFi IOL models. Ganesh et al. [25] evaluated the outcomes at 6 months of the partial-DOFi IOL Symfony (Johnson & Johnson Vision), obtaining a mean binocular DCIVA value of 0.104 ± 0.08 logMAR. Similarly, Schojai et al. [22] obtained mean monocular 3-month postoperative UIVA of 0.05 ± 0.08 logMAR in a sample of eyes implanted with the Symfony IOL. Zeilinger et al. [24] reported mean 3-month postoperative UIVA and DCIVA of 0.11 ± 0.10 and 0.14 ± 0.10 logMAR with another partial-DOFi IOL (Vivity, Alcon). Tahmaz et al. [26] also observed significantly better binocular DCIVA with the partial-DOFi IOL LuxSmart versus two monofocal IOLs, noting that 68% of eyes achieved monocular DCIVA better than 20/23. In our study, postoperative DCIVA (3 months) was ≤0.20 logMAR in 100% of DOFi-implanted eyes versus 23% of monofocal-implanted eyes, further underscoring the partial-DOFi IOL’s advantage in intermediate vision. Janekova et al. [23] conducted an intraindividual comparative study, implanting an enhanced partial-DOFi IOL (Eyhance, Johnson & Johnson Surgical Vision) in one eye and a monofocal IOL in the fellow eye. They reported mean UIVA (66 cm) of 0.32 ± 0.19 logMAR (partial-DOFi) versus 0.45 ± 0.16 logMAR (monofocal). Similarly, Bova et al. [19] found worse DCIVA with a monofocal IOL compared to a partial-DOFi IOL (Isopure, BVI Medical), with the partial-DOFi achieving 0.23 ± 0.07 logMAR.

Significant differences in DCNVA were observed between the monofocal and partial-DOFi IOL groups. The mean 3-month postoperative monocular DCNVA was 0.31 ± 0.09 logMAR with the PRoF IOL compared to 0.51 ± 0.10 logMAR with the monofocal IOL (*p* < 0.001). The DCNVA achieved with the PRoF IOL was comparable [27] or superior [20,21,28,29] to other partial-DOFi IOL models. Schojai et al. [22] obtained mean monocular 3-month postoperative UNVA of 0.21 ± 0.08 logMAR in eyes implanted with the Symfony IOL. Likewise, for this same partial-DOFi IOL, Ganesh et al. [25] found a mean 6-month postoperative binocular DCNVA value of 0.216 ± 0.10 logMAR. In contrast, Zeilinger et al. [24] reported a somewhat worse mean 3-month postoperative DCNVA (0.35 ± 0.16 logMAR) in eyes with the partial-DOFi IOL Vivity (Alcon). Iradier et al. [21] reported a mean 3-month postoperative DCNVA of 0.37 ± 0.36 logMAR with the Synthesis Plus IOL (Cutting Edge). Similarly, Lesieur and Dupeyre [28] found binocular DCNVA values of 0.08 ± 0.09, 0.29 ± 0.13, and 0.40 ± 0.17 logMAR for the Lucidis, Synthesis Plus, and Isopure IOLs, respectively. In our study, 66% of partial-DOFi-implanted eyes achieved a monocular DCNVA ≤0.30 logMAR, compared to only 4% in the monofocal group. These results align with prior findings: Bernabeu-Arias et al. [29] reported that 41.94% of Isopure IOL patients attained binocular DCNVA ≥20/40, while Ruiz-Mesa et al. [30] observed this threshold in 72.73% of LuxSmart IOL patients. Future research should explore whether micromonovision (targeting slight myopia in the non-dominant eye) could further enhance near vision with this partial-DOFi IOL, as demonstrated with other models [31,32]. For example, Van Amelsfort et al. [32] showed that mini-monovision with the partial-DOFi Vivity IOL (Alcon) improved near vision and spectacle independence.

The defocus curve results demonstrate the superior range of vision provided by the partial-DOFi IOL compared to the monofocal IOL, with significantly better distance-corrected visual acuity at all defocus levels from −1.50 to −4.00 D (*p* < 0.05). As shown in Figure 3, the PRoF IOL maintained a mean binocular distance-corrected visual acuity ≤ 0.30 logMAR across a wider defocus range (+0.50 D to −2.50 D) than the monofocal IOL (+0.50 D to −1.50 D). These findings align with prior studies. Obergozo et al. [20] reported a similar functional range (+0.50 D to −2.00 D) for the LuxSmart partial-DOFi IOL. In contrast, Iradier et al. [21] observed a narrower range (+1.00 D to −1.50 D) with the Synthesis Plus IOL, suggesting variability among partial-DOFi designs. The partial-DOFi IOL evaluated in the current series has shown to provide a DoF at 0.2 logMAR (2.4 D) compatible with the subcategory of extend partial-DOFi according to the ESCRS functional classification system [2,3]. Notably, a significant superiority in depth of focus was found for the evaluated partial-DOFi IOL over the control IOL, even though the control was an aspheric monofocal lens. Such lenses may themselves provide limited pseudoaccommodation through spherical aberration control, pupil interaction, and residual astigmatism. Future studies should compare this partial-DOF IOL with other monofocal models, as this may reveal even greater depth-of-focus benefits.

Patient-reported outcomes, including photic phenomena and vision-related task difficulty, were also evaluated. The QoV questionnaire revealed comparable levels of photic phenomena between the monofocal and partial-DOFi IOL groups, with only minor reports of starbursts in a few cases. This finding contrasts with prior studies reporting increased photic phenomena with certain partial-DOFi IOL models [33], suggesting that the evaluated partial-DOFi IOL preserves visual quality without inducing additional dysphotopsias. The Catquest questionnaire demonstrated significant advantages for the partial-DOFi IOL group in daily activities, including reduced difficulty reading newspapers, improved visibility of prices while shopping, and enhanced performance of hobbies and needlework. These results confirm that the superior DCIVA and near DCNVA provided by the partial-DOFi IOL translates to tangible benefits in patients’ daily lives. However, no significant difference in overall satisfaction was observed between groups, underscoring the dominant role of distance vision in postoperative satisfaction—a finding consistent with the meta-analysis by Karam et al. [34], which reported similar satisfaction rates between trifocal and partial-DOFi IOLs despite differences in near vision. This finding can also be influenced in our study by the lack of randomization in the assignment to the partial-DOFI and monofocal groups and, thus, the visual expectancies of patients included in each group may differ significantly.

This study has several limitations. First, it was conducted at a single center with a moderate sample size, which may limit the generalizability of the findings. Second, the follow-up period was limited to three months; while longer than most studies and sufficient to assess refractive stability and early visual outcomes, longer-term data would be valuable to evaluate neuroadaptation processes and patient satisfaction over time. Third, a statistically significant age difference between groups presents an additional limitation. Age-related pupil miosis and its effect on pupil-mediated pseudoaccommodation could have biased the depth-of-focus results. However, as shown in Table 1, no significant differences in photopic or mesopic pupil sizes were found between the groups. Therefore, the influence of this factor appears to be negligible.

## 5. Conclusions

In conclusion, the partial-DOFi Extended TECNIS PureSee IOL demonstrates superior intermediate and near visual performance compared to a monofocal IOL, while maintaining equivalent distance vision and visual quality. These functional advantages translate into measurable improvements in patients’ daily activities, particularly for vision-dependent tasks such as reading and hobbies. By combining extended depth of focus with minimal photic phenomena, the TECNIS PureSee IOL represents a compelling option for patients seeking reduced spectacle dependence without compromising optical quality. In addition, the TECNIS PureSee IOL meets both the ANSI standards for EDOF IOLs and the criteria of partial-DOFi-extended IOLs according to the ESCRS functional classification system and therefore it should be classified this way.

## Figures and Tables

**Table 1:** Preoperative biometric and corneal topographic data of the sample evaluated. Abbreviations: Partial-DOFi, partial increase in depth of focus; D, diopter; UDVA, uncorrected-distance visual acuity; SE, spherical equivalent; CDVA, corrected-distance visual acuity; K1, flattest keratometric reading; K2, steepest keratometric reading; CCT, central corneal thickness; AXL, axial length; ACD, anterior chamber depth; WTW, white-to-white corneal diameter; LT, lens thickness; IOP, intraocular pressure.

Mean (SD) Median (Min to Max)	Monofocal Group (26 Eyes)	Partial-DOFi Group (29 Eyes)	*p*-Value
Age (years)	75.4 (5.2) 76.0 (67.0 to 89.0)	70.5 (7.8) 71.0 (56.0 to 87.0)	0.009
LogMAR UDVA	0.70 (0.64) 0.40 (0.10 to 3.00)	0.75 (0.46) 0.52 (0.20 to 1.60)	0.369
Manifest sphere (D)	−1.37 (2.94) −1.00 (−13.00 to 1.75)	−1.42 (3.60) 0.00 (−10.00 to 2.75)	0.493
Manifest cylinder (D)	−0.59 (0.50) −0.50 (−2.00 to 0.00)	−0.79 (0.85) −0.75 (−2.50 to 0.00)	0.635
SE (D)	−1.66 (2.95) −1.13 (−13.00 to 1.75)	−1.82 (3.62) −0.75 (−10.37 to 2.75)	0.729
LogMAR CDVA	0.17 (0.13) 0.10 (0.00 to 0.44)	0.24 (0.22) 0.20 (0.00 to 0.70)	0.333
K1 (D)	43.78 (1.46) 43.78 (41.20 to 46.46)	43.00 (1.80) 42.65 (40.06 to 46.46)	0.087
K2 (D)	44.56 (1.41) 44.60 (41.65 to 46.91)	43.79 (1.82) 43.56 (40.98 to 47.04)	0.088
CCT (µm)	551.19 (38.51) 540.00 (479.00 to 627.00)	544.48 (30.07) 542.00 (458.00 to 587.00)	0.833
AXL (mm)	24.07 (1.30) 23.75 (21.89 to 28.15)	24.30 (1.81) 24.02 (22.07 to 29.58)	0.833
ACD (mm)	3.14 (0.45) 3.15 (2.49 to 4.08)	3.11 (0.42) 3.17 (2.18 to 3.79)	0.764
WTW (mm)	12.04 (0.42) 12.05 (11.30 to 12.70)	12.03 (0.42) 12.00 (11.40 to 12.90)	0.917
LT (mm)	4.65 (0.36) 4.74 (4.05 o 5.22)	4.63 (0.44) 4.56 (3.78 to 5.97)	0.869
Photopic pupil size (mm)	2.93 (0.43) 2.97 (2.12 to 3.68)	2.97 (0.50) 2.93 (2.08 to 3.84)	0.730
Mesopic pupil size (mm)	4.35 (0.83) 4.09 (2.92 to 5.94)	4.40 (0.54) 4.31 (3.36 to 5.62)	0.782

**Table 2:** Postoperative visual and refractive data in the three groups of the sample evaluated. Abbreviations: Partial-DOFi, partial increase in depth of focus; D, diopter; UDVA, uncorrected-distance visual acuity; CDVA, corrected-distance visual acuity; DCIVA, distance-corrected intermediate visual acuity; DCNVA, distance-corrected near visual acuity.

Mean (SD) Median (Min to Max)	Monofocal Group (26 Eyes)	Partial-DOFi Group (29 Eyes)	*p*-Value
1-week postoperative visit			
Monocular logMAR UDVA	0.05 (0.09) 0.00 (−0.10 to 0.30)	0.07 (0.08) 0.10 (−0.08 to 0.30)	0.373
Sphere (D)	0.03 (0.15) 0.00 (−0.25 to 0.25)	−0.10 (0.25) 0.00 (−0.75 to 0.50)	0.014
Cylinder (D)	−0.13 (0.26) 0.00 (−0.75 to 0.00)	−0.15 (0.25) 0.00 (−1.00 to 0.00)	0.728
SE (D)	−0.07 (0.21) −0.12 (−0.50 to 0.37)	−0.23 (0.22) −0.25 (−1.00 to 0.00)	0.020
Monocular logMAR CDVA	0.00 (0.06) 0.00 (−0.10 to 0.20)	0.02 (0.06) 0.00 (−0.08 to 0.15)	0.170
1-month postoperative visit			
Monocular logMAR UDVA	0.03 (0.07) 0.00 (−0.08 to 0.20)	0.04 (0.07) 0.00 (−0.08 to 0.20)	0.243
Sphere (D)	0.06 (0.19) 0.00 (−0.25 to 0.50)	−0.02 (0.20) 0.00 (−0.50 to 0.50)	0.180
Cylinder (D)	−0.20 (0.32) −0.13 (−1.00 to 0.00)	−0.17 (0.20) 0.00 (−0.50 to 0.00)	0.900
SE (D)	−0.04 (0.21) 0.00 (−0.37 to 0.50)	−0.10 (0.19) −0.13 (−0.62 to 0.25)	0.522
Monocular logMAR CDVA	−0.02 (0.04) 0.00 (−0.10 to 0.05)	0.00 (0.05) 0.00 (−0.08 to 0.10)	0.069
Monocular logMAR DCIVA	0.30 (0.07) 0.30 (0.10 to 0.40)	0.11 (0.03) 0.10 (0.10 to 0.20)	<0.001
Monocular logMAR DCNVA	0.48 (0.11) 0.49 (0.10 to 0.70)	0.29 (0.08) 0.30 (0.10 to 0.40)	<0.001
3-month postoperative visit			
Monocular logMAR UDVA	0.03 (0.08) 0.00 (−0.08 to 0.30)	0.05 (0.10) 0.00 (−0.08 to 0.30)	0.419
Sphere (D)	0.08 (0.26) 0.00 (−0.25 to 0.75)	−0.04 (0.28) 0.00 (−0.50 to 0.50)	0.156
Cylinder (D)	−0.34 (0.42) −0.25 (−1.75 to 0.00)	−0.13 (0.21) 0.00 (−0.75 to 0.00)	0.035
SE (D)	−0.09 (0.24) −0.13 (−0.62 to 0.50)	−0.11 (0.29) 0.00 (−0.62 to 0.50)	0.925
Monocular logMAR CDVA	−0.03 (0.04) 0.00 (−0.08 to 0.00)	−0.03 (0.05) 0.00 (−0.08 to 0.10)	0.642
Monocular logMAR DCIVA	0.29 (0.08) 0.30 (0.10 to 0.40)	0.10 (0.06) 0.10 (0.00 to 0.30)	<0.001
Monocular logMAR DCNVA	0.51 (0.10) 0.49 (0.30 to 0.70)	0.31 (0.09) 0.30 (0.00 to 0.40)	<0.001

**Table 3:** Summary of postoperative Rasch-calibrated scoring obtained with the Catquest-9SF questionnaire in the two groups of the sample evaluated in the current study.

Mean (SD) Median (Min to Max)	Monofocal Group (26 Eyes)	Partial-DOFi Group (29 Eyes)	*p*-Value
Item A: Do you experience that your present vision gives you difficulties in any way in your daily life?	−2.83 (1.37) −3.98 (−3.98 to −1.26)	−3.79 (0.70) −3.98 (−3.98 to −1.26)	0.002
Item B: Are you satisfied or dissatisfied with your present vision?	−2.22 (0.89) −2.53 (−2.53 to 0.20)	−2.53 (0.00) −2.53 (−2.53 to −2.53)	0.062
Item C1: Do you have difficulty with reading text in the newspaper because of your vision?	−0.18 (1.69) −0.23 (−4.18 to 3.02)	−3.05 (1.36) −4.18 (−4.18 to −1.46)	<0.001
Item C2: Do you have difficulty with recognizing faces of people you meet because of your vision?	−3.63 (0.00) −3.63 (−3.63 to −3.63)	−3.63 (0.00) −3.63 (−3.63 to −3.63)	1.000
Item C3: Do you have difficulty with seeing prices of goods when shopping because of your vision?	−1.70 (1.74) −1.72 (−4.44 to 2.76)	−4.06 (0.95) −4.44 (−4.44 to −1.72)	<0.001
Item C4: Do you have difficulty with seeing to walk on uneven ground because of your vision?	−3.46 (0.89) −3.77 (−3.77 to −1.05)	−3.77 (0.00) −3.77 (−3.77 to −3.77)	0.062
Item C5: Do you have difficulty with seeing to do needlework and handicraft because of your vision?	−0.88 (2.23) −0.65 (−3.37 to 3.83)	−2.90 (1.05) −3.37 (−3.37 to −0.65)	<0.001
Item C6: Do you have difficulty with reading text on television because of your vision?	−3.83 (1.41) −4.59 (−4.59 to −1.31)	−4.36 (0.85) −4.59 (−4.59 to −1.31)	0.092
Item C7: Do you have difficulty seeing to carry out a preferred hobby because of your vision?	−4.57 (0.90) −4.95 (−4.95 to −2.51)	−4.95 (0.00) −4.95 (−4.95 to −4.95)	0.030

## Data Availability

The data that support the findings of this study are available from the first author, HN, upon reasonable request.
